# Identification of potential autophagy-associated lncRNA in prostate cancer

**DOI:** 10.18632/aging.202997

**Published:** 2021-05-10

**Authors:** Jun Li, Hong Du, Wenqiang Chen, Mingxing Qiu, Peng He, Zhiwei Ma

**Affiliations:** 1 Department of Urology, Sichuan Provincial People’s Hospital, University of Electronic Science and Technology of China, Chengdu, China

**Keywords:** prostate cancer, lncRNA, gene signature, ceRNA, autophagy

## Abstract

Background: Long non-coding RNAs (lncRNAs) have been linked to autophagy. It is urgent to identify and assess the hub autophagy-associated lncRNA in prostate cancer.

Methods: Differentially expressed lncRNAs associated with autophagy were identified in prostate cancer based on The Cancer Genome Atlas Prostate Adenocarcinoma (TCGA-PRAD) data. An autophagy-mediated competing endogenous RNA network was constructed to screen for autophagy-associated lncRNA, and the preselected lncRNAs were further validated using Gene Expression Omnibus (GEO) datasets. Furthermore, a prognostic lncRNA signature was established and assessed. Additionally, Gene Set Enrichment Analysis (GSEA) revealed the underlying molecular mechanisms.

Results: Using a competing endogenous RNA network, 66 differentially expressed lncRNAs associated with autophagy were identified, and the differential expression of 7 lncRNAs were verified using the TCGA-PRAD, GSE21034, and GSE94767 datasets. Additionally, a lncRNA signature associated with autophagy, including MKNK1-AS1 and INE1, was identified as an independent indicator of survival with a C-index of 0.882. The GSEA analysis indicated that several autophagy-related signaling pathways were enriched in different risk groups.

Conclusions: The lncRNAs associated with autophagy were identified, and a prediction model was developed that could be used as a prognostic predictor for prostate cancer, indicating the critical role of lncRNA in the regulation of prostate cancer autophagy regulation.

## INTRODUCTION

Prostate cancer (PCa) is a significant health concern, with estimated 359 000 deaths globally in 2018 [1]. PCa is a highly heterogeneous disease, and the prognosis for patients with PCa varies dramatically [2]. Prostate cancer is considered an indolent tumor. For early-stage PCa, there are many effective treatment options such as surgery, androgen deprivation therapy, chemotherapy, and radiotherapy. However, 25% of patients will still suffer from recurrence and metastasis, leading to the development of a highly aggressive castration-resistant PCa with poor survival and limited treatment strategies [3]. Therefore, it is critical to identify high-risk PCa and implement appropriate treatment strategies as early as possible to prolong survival in PCa patients. In clinical practice, patients with different Gleason scores or PSA levels at the same stage may have different survival outcomes due to the heterogeneity of PCa [4]. Consequently, it is desirable to identify more effective diagnostic and prognostic biomarkers for PCa.

Autophagy is an evolutionarily conserved self-degradative process in which cytoplasmic proteins and organelles are eradicated and reused to maintain a metabolic adaptation during synthesis, degradation, and subsequent cycles of cellular product [5]. Autophagy is disrupted in several diseases, including neurodegenerative diseases, inflammatory diseases, cardiovascular diseases, and cancer [6]. Autophagy has been implicated in cancer in numerous studies, and the effective targeting of autophagy may be an underlying therapeutic strategy in advanced cancer [7]. Death-associated protein kinase 3 (DAPK3), a novel autophagy regulator, may phosphorylate ULK1 and enable it to suppress gastric cancer progression [8]. CircMUC16 induced autophagy and accelerated epithelial ovarian cancer progression by interacting directly with miR-199a and ATG13 [9]. In addition, a growing number of studies have demonstrated that impaired autophagy is linked to prostate cancer [10]. Phospholipase C epsilon (PLCɛ) modulates the AR signaling activities in PCa cell lines through the degradation mechanism that is dependent on autophagy [11]. MicroRNA-381 reduces the RELN-mediated PI3K/AKT pathway activity, which inhibits cell proliferation and promotes apoptosis and autophagy in PCa cells [12]. These findings support the correlation between autophagy and PCa. However, the potential molecular mechanisms of action associated with autophagy in PCa are still unknown and unexplored.

Numerous studies have examined the critical functions of lncRNA in the initiation and progression of cancer [13]. Additionally, some lncRNAs are involved in the occurrence and progression of PCa [14]. For example, dysregulations of KCNQ1OT1 have been discovered in PCa cells and tissues. Enhanced lncRNA KCNQ1OT1 expression sponged miR-15a to facilitate immune escape and progression by improving the expression of PD-L1 in PCa [15]. LINC00173 functioned as an oncogene in PCa via the LINC00173/miR-338-3p/Rab25 pathways [16]. These findings revealed a strong connection between lncRNA and PCa. However, the study of the lncRNA in PCa is insufficient, and it is urgent to discover lncRNAs that may play a vital role in PCa.

In this study, we aim to establish a network of ceRNA to screen for lncRNA associated with autophagy as novel biomarkers in PCa and to construct a lncRNA signature associated with autophagy to predict the prognosis of PCa patients. It is valuable to provide new information about the clinical application of the lncRNA associated with autophagy in PCa.

## RESULTS

### DEmRNAs, DEmiRNAs, and DElncRNAs in PCa

**Figure 1:**
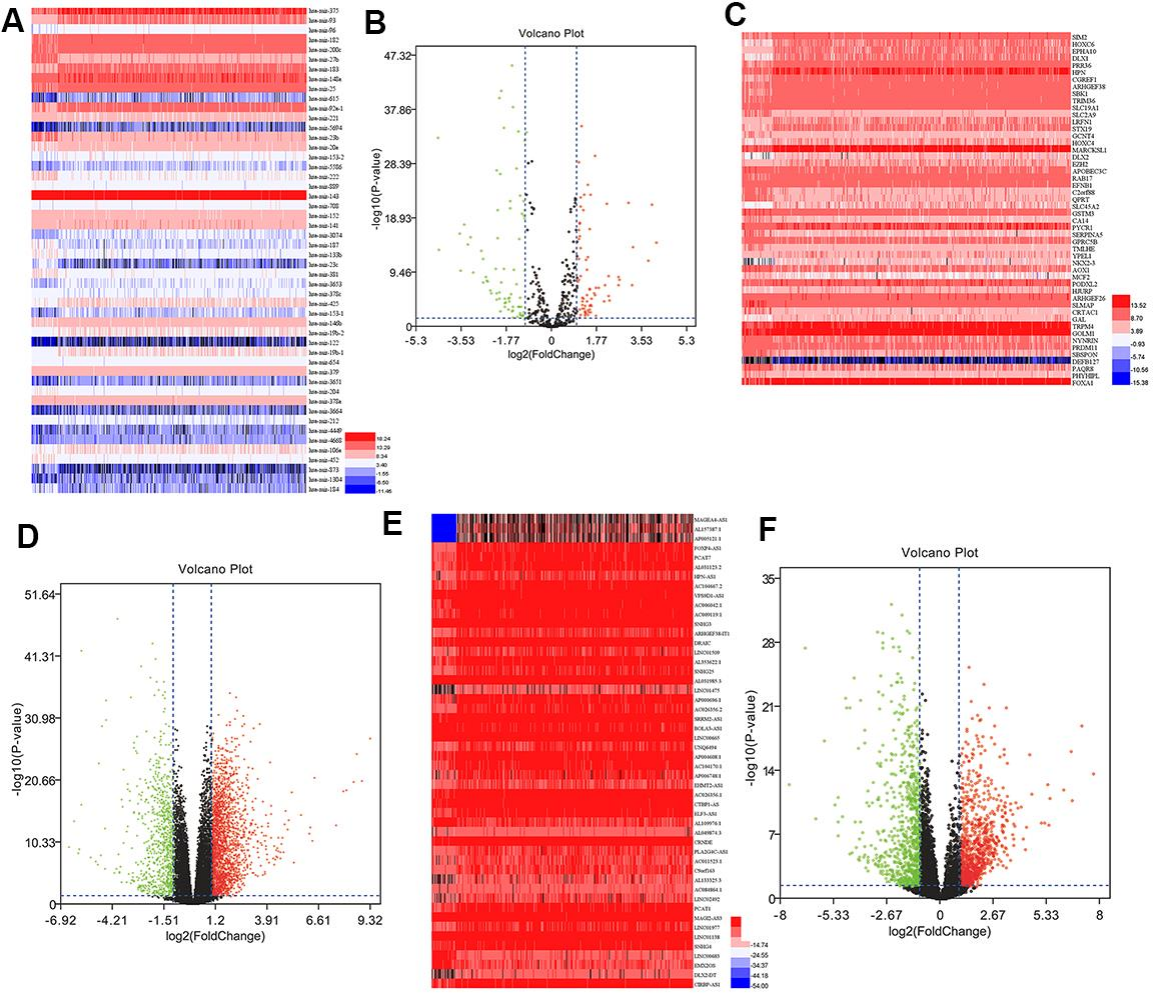
**DEmiRNAs, DEmRNAs, and DElncRNAs in prostate cancer.** (**A**) Heatmap of top 50 DEmiRNAs. (**B**) Volcano plot of DEmiRNAs. (**C**) Heatmap of top 50 DEmRNAs. (**D**) Volcano plot of DEmRNAs. (**E**) Heatmap of top 50 DElncRNAs. (**F**) Volcano plot of DElncRNAs. FC, fold change; DEmiRNAs, differently expressed miRNAs; DEmRNAs, differently expressed mRNAs; DElncRNAs, differently expressed lncRNAs.

Differential expression analysis of PCa and adjacent tissues identified 149 miRNAs (71 upregulated and 78 downregulated), 2947 mRNAs (2108 upregulated and 839 downregulated), and 1932 lncRNAs (1028 upregulated and 904 downregulated). Subsequently, the volcano plots were used to visualize DEmiRNA, DEmRNA, and DElncRNA (Figure 1). The top 50 DEmiRNAs, DEmRNAs, and DElncRNAs were displayed using heatmaps in Figure 1. DEmiRNAs, DEmRNAs, and DElncRNAs could distinguish between PCa tissues and normal tissues.

### Establishment of an autophagy-associated ceRNA regulatory network

**Figure 2:**
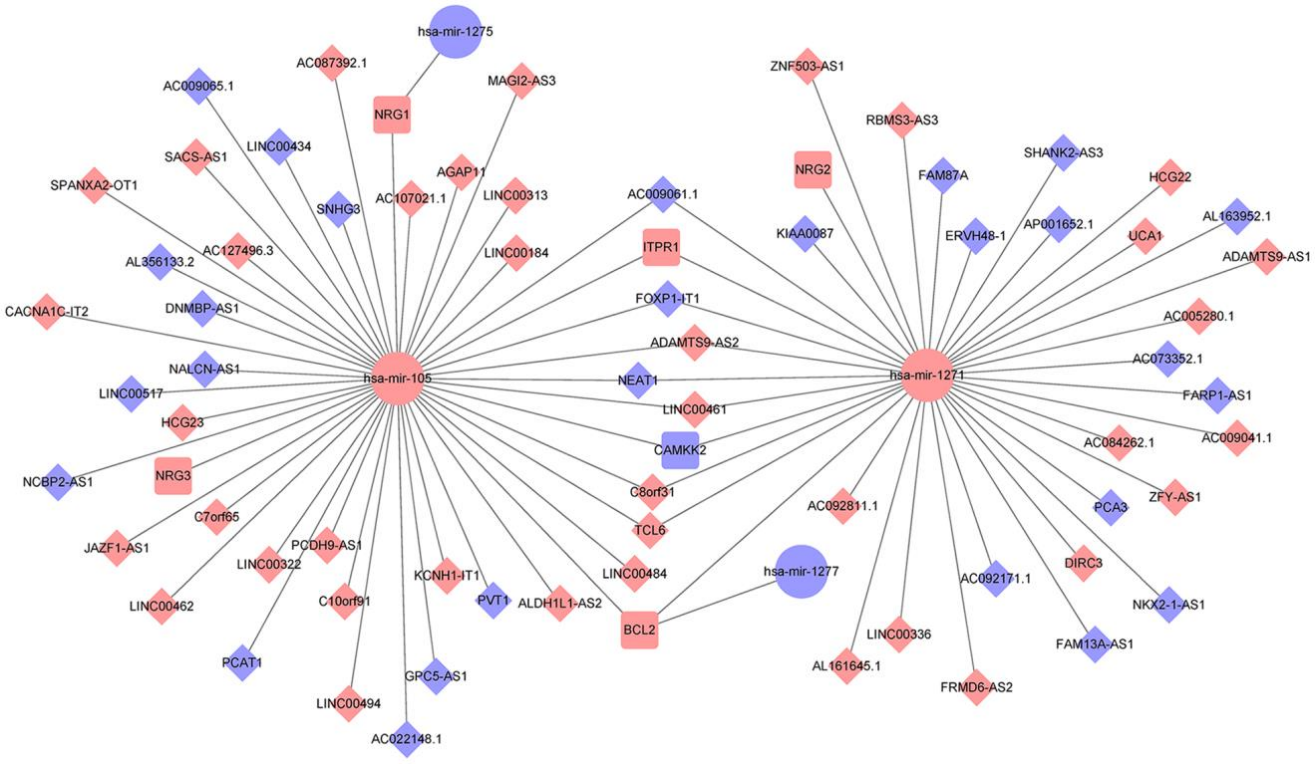
**Establishment of an autophagy-associated ceRNA regulatory network in prostate cancer.** The blue and pink nodes exhibited reduced and enhanced expression of RNAs. Diamonds represent lncRNAs, ellipses represent miRNAs, rectangles represent mRNAs, and gray edges represent interactions among the lncRNAs-miRNAs and mRNAs.

We also established a ceRNA regulatory network to predict the critical lncRNA associated with autophagy in PCa. We identified fourteen autophagy-related DEmRNAs (ATG9B, BCL2, BIRC5, CAMKK2, CDKN2A, HSPB8, ITGB4, ITPR1, NKX2-3, NRG1, NRG2, NRG3, TMEM74, TP63) as candidates from the screened 2947 DEmRNAs and 232 genes associated with autophagy. The TargetScan, miRTarBase, and miRanda databases were applied to predict the specific DEmiRNAs that interacted with the fourteen candidate mRNAs. Afterward, 66 DElncRNAs interacting with the four selected miRNAs were identified using the miRcode database. Finally, 66 lncRNAs, four miRNAs, and six mRNAs were involved in the autophagy-mediated ceRNA network based on miRNA-mRNA and lncRNA-miRNA interactions (Figure 2).

### Verification of the identified lncRNAs in the GEO database

**Figure 3:**
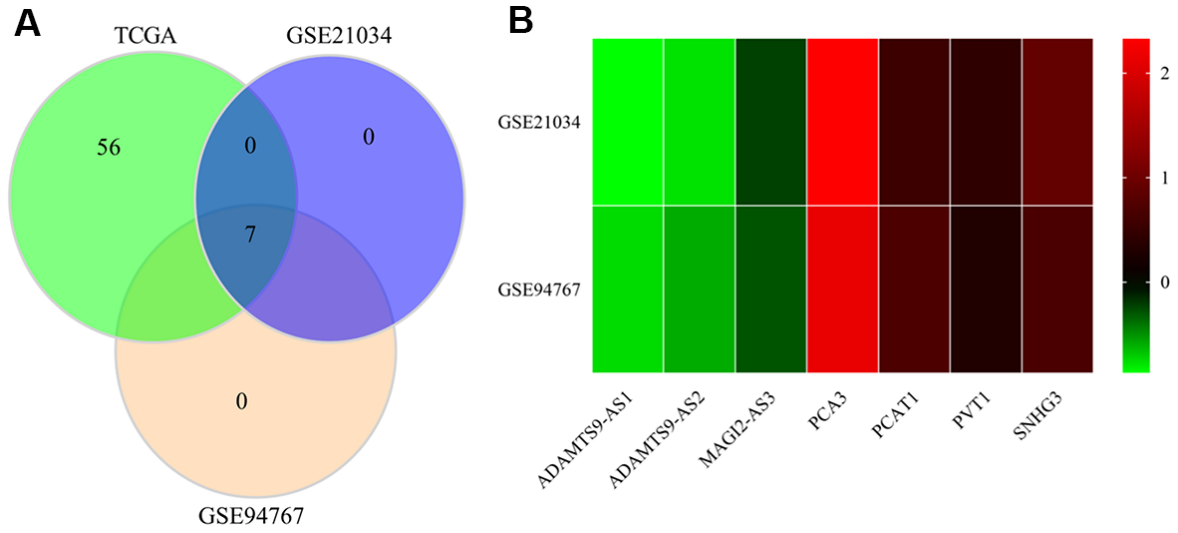
**Verification of the identified lncRNAs in GEO datasets.** (**A**) Venn diagrams of differently expressed lncRNAs in the TCGA, GSE21034, and GSE94767 datasets. (**B**) Heatmap of differently expressed lncRNAs in the TCGA, GSE21034, and GSE94767 datasets.

We further selected the GSE21034 and GSE94767 datasets from the GEO database to verify the differential expression of 66 autophagy-linked lncRNAs filtered by the autophagy-mediated ceRNA network. As illustrated by Figure 3A, 3B, only 7 (ADAMTS9-AS1, ADAMTS9-AS2, MAGI2-AS3, PCA3, PCAT1, PVT1, SNHG3) of the 66 autophagy-related lncRNAs in the TCGA, GSE21034, and GSE94767 datasets showed abnormal expression levels in PCa tissues compared to normal prostate tissue.

### Identification of the autophagy-associated lncRNA signature

**Figure 4:**
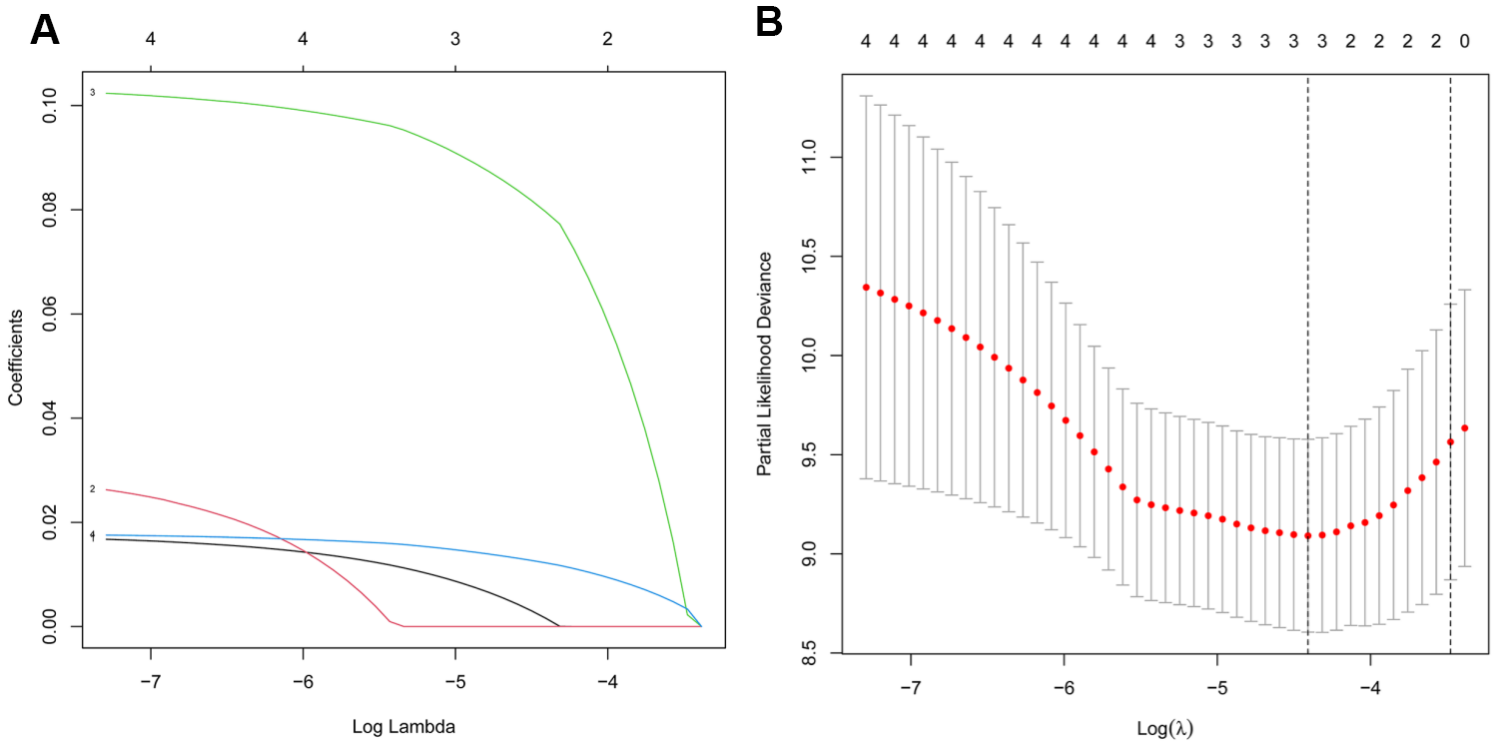
**Screening of the autophagy-associated lncRNA in prostate cancer by LASSO model.** (**A**) Plots of the cross-validation error rates. (**B**) LASSO coefficient profiles of the prognostic lncRNAs.

232 autophagy-related genes were acquired from HADb and 1357 autophagy-related lncRNAs were identified using the Pearson correlation coefficients between the expression of lncRNAs and autophagy-related genes with |R| > 0.5 and P < 0.05 as screening criteria. Additionally, univariate Cox regression and Kaplan-Meier analysis were used to obtain AC008760.1, AC134775.1, MKNK1-AS1, and INE1. LASSO regression was further used to identify three autophagy-associated lncRNAs (AC008760.1, MKNK1-AS1, and INE1) in Figure 4. Subsequently, multivariate Cox regression revealed that two of the three autophagy-associated lncRNAs, MKNK1-AS1 and INE1, were candidates for the prognostic signature derived from the lowest Akaike information criterion (AIC) value (Table 1). MKNK1-AS1 and INE1, two autophagy-associated lncRNAs in the prognostic signature, were both found to be adverse prognostic factors with a hazard ratio (HR)>1. In the lncRNA signature associated with autophagy, the following risk score formula for patients with PCa was as follows: risk score = 0.1277 * expression level of MKNK1-AS1 +0.0186 * expression level of INE1.

**Table 1:** Akaike information criterion for the models.

**Model**	**Prognostic signature combination**	**AIC**
1	AC008760.1 + MKNK1-AS1 + INE1	62.08
2	MKNK1-AS1 + INE1	61.7

### Assessment of the autophagy-associated lncRNA signature

**Figure 5:**
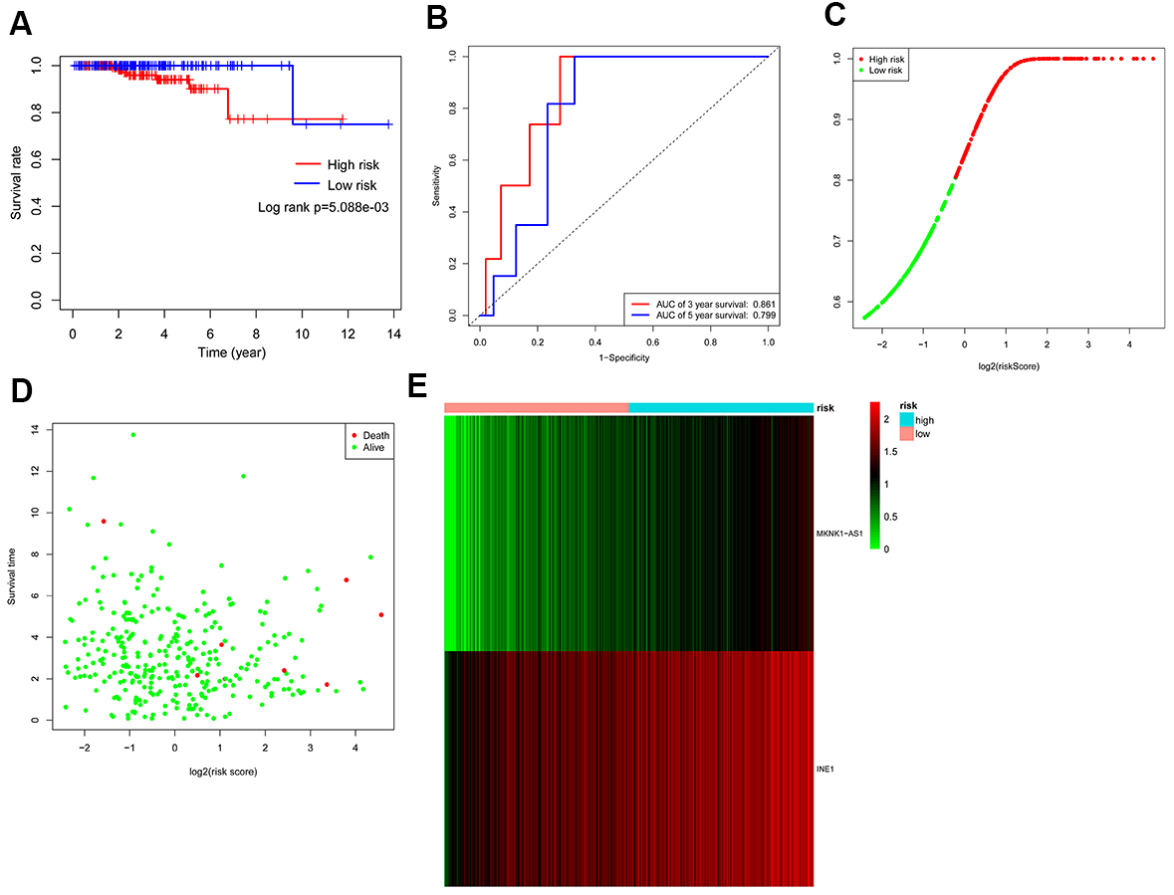
**Assessment of the autophagy-associated lncRNA signature.** (**A**) Kaplan-Meier analysis of prostate cancer patients stratified by the median risk score. (**B**) ROC analysis of the sensitivity and specificity of the survival for the risk score. (**C**) Risk score distributions between the high-risk group and the low-risk group. (**D**) The scatter plot exhibited the associations between risk score, survival status, and survival time. (**E**) Heatmap of two autophagy-related lncRNA expressions.

PCa patients were divided into low-risk and high-risk groups for autophagy-associated lncRNA signature analysis based on the median risk score. Furthermore, the survival curve indicated that patients with high-risk scores had a shorter survival time (Figure 5A). As shown in Figure 5B, the time-dependent ROC curve analysis exhibited that the AUC value of the ROC curve for the autophagy-associated lncRNA signature corresponding to three years and five years of survival was 0.861 and 0.799, respectively. The results indicated that the autophagy-related lncRNA signature might be used to predict prognostic value. Additionally, the C-index of the prognostic signature was 0.882 (95% CI: 0.800-1.055), indicating that the risk score had a reliable and accurate diagnostic impact on PCa diagnosis. As shown in Figure 5C, the risk score distributions for PCa patients were categorized and plotted based on the risk score. Besides, the scatter plot exhibited the relationships between risk score, survival status, and survival time in PCa patients (Figure 5D). Moreover, the heatmap revealed two autophagy-associated lncRNAs were expressed differently in the high-risk and low-risk PCa patients (Figure 5E). These findings suggested that the autophagy-associated lncRNA signature appeared to be useful in the diagnosis and prognosis of PCa.

### Independent predictive factor in PCa

**Figure 6:**
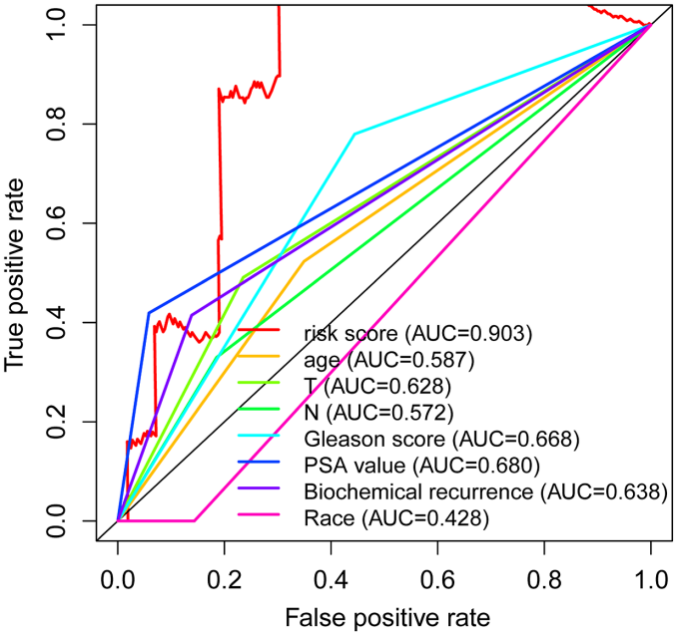
**Independent predictive factor in prostate cancer patients.** Receiver operating characteristic (ROC) curve analysis shows the prognostic accuracy of clinicopathological parameters such as age, T stage, N stage, Gleason score, PSA value, biochemical recurrence, Race, and autophagy-related lncRNA prognostic risk score.

Univariate Cox regression exhibited that the T stage (P =0.049) and risk score (P < 0.001) were significantly associated with survival in the PCa, with the HR of risk score being 1.173 (95% CI: 1.079–1.275) in Table 2. Furthermore, multivariate Cox regression further revealed that the risk score (P =0.010) was significantly associated with PCa survival, with a HR of 1.145 (95% CI: 1.033–1.270) (Table 2). Additionally, the ROC curve analysis revealed that the AUC value of the autophagy-associated lncRNA signature was 0.903, which was higher than the AUC values for age (AUC = 0.587), T stage (AUC= 0.628), N stage (AUC=0.572), Gleason score (AUC=0.668), PSA value (AUC= 0.680), biochemical recurrence (AUC=0.638) and Race (AUC=0.428) in Figure 6. In addition, we further analyzed the relationship between autophagy-associated lncRNA signature and clinical features (Table 3), and the results revealed that the Gleason score was significantly related to the autophagy-associated lncRNA signature (P =0.008). Overall, these results indicated that the autophagy-related lncRNA signature was an independent prognostic factor for PCa patients.

**Table 2:** Univariate and multivariate Cox regression of clinicopathologic factors for survival in prostate cancer.

**Clinical characteristics**	**HR**	**P-value**
**Univariate Cox regression**		
Age (<65 vs.≥65)	3.799 (0.897-16.081)	0.070
T stage (T1+T2 vs. T3+T4)	4.526 (1.008-20.321)	0.049
N stage (N0 vs. N1)	4.376 (0.881-21.729)	0.071
Gleason score (≤7 vs. >7)	8.130 (0.970-68.145)	0.053
PSA value (<4ng/ml vs. ≥4ng/ml)	3.982 (0.464-34.192)	0.208
Biochemical recurrence (NO vs. YES)	3.718 (0.830-16.663)	0.086
Race (White vs. Other)	0.802 (0.132-4.867)	0.811
RiskScore (Low vs. High)	1.173 (1.079-1.275)	<0.001
**Multivariate Cox regression**		
T stage (T1+T2 vs. T3+T4)	1.979 (0.311-12.591)	0.470
RiskScore (Low vs. High)	1.145 (1.033-1.270)	0.010

**Table 3:** The association between the autophagy-associated lncRNA signature and clinical features.

**Clinical**	**n**	**Mean**	**SD**	**t**	**P**
age (<65)	208	1.696	2.833	-0.99796	0.319
age (≥65)	114	2.028	2.867		
T stage (T1+T2)	244	1.688	2.444	-1.12381	0.264
T stage (T3+T4)	78	2.206	3.831		
N stage(N0)	261	1.681	2.478	-1.29826	0.198
N stage(N1)	61	2.38	4.035		
Gleason score (≤7)	176	1.411	2.055	-2.69175	0.008
Gleason score (>7)	146	2.299	3.519		
PSA value (<4ng/ml)	300	1.792	2.729	-0.34797	0.731
PSA value (≥4ng/ml)	22	2.108	4.195		
Biochemical recurrence (NO)	275	1.646	2.422	-1.70034	0.095
Biochemical recurrence (YES)	47	2.793	4.514		
Race (White)	277	1.702	2.711	-1.45618	0.151
Race (Other)	45	2.501	3.519		

### Gene set enrichment analysis

**Figure 7:**
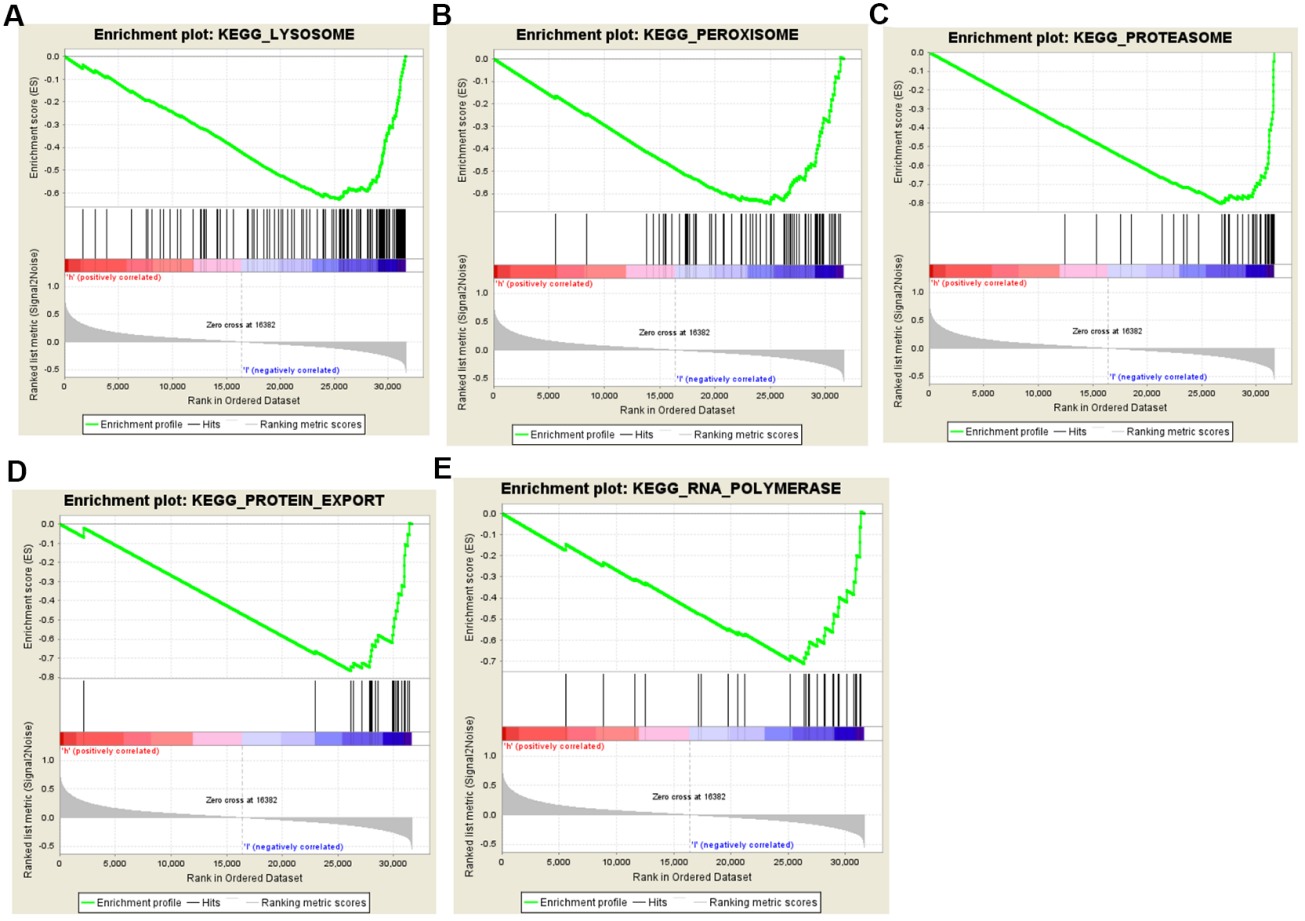
**Gene set enrichment analysis (GSEA) of high-risk and low-risk prostate cancer patients based on the autophagy-related lncRNA prognostic signature.** The GSEA analysis showed that samples with low-risk patients enriched in (**A**) lysosome, (**B**) peroxisome, (**C**) proteasome, (**D**) protein export, and (**E**) RNA polymerase pathways.

To identify the potential enhanced signaling pathway between the high-risk and low-risk groups, we performed the Gene Set Enrichment Analysis (GSEA). As shown in Figure 7, peroxisome (NES=--2.191, P=0.002), lysosome (NES=--2.110, P<0.001), RNA polymerase (NES=-2.062, P<0.001), proteasome (NES=-1.962, P<0.001), and protein export (NES=-1.856, P=0.002) were all significantly altered in the low-risk group, indicating that autophagy was strongly related to PCa. These results provided a new horizon for the development of personalized treatments for PCa patients with different risk scores.

## DISCUSSION

The occurrence rate of prostate cancer, an epithelial malignancy that occurs in the prostate gland, has remained elevated in recent years [17]. Prostate cancer is one of the malignant tumors with the highest five-year survival rate. Early patients encounter challenges in diagnosis, and advanced patients who are predisposed to relapse have little medical choices [18]. Besides, autophagy is involved in the malignant progression of a variety of tumors, including PCa [19]. As a result, autophagy-related biomarkers may be used as diagnostic and therapeutic targets for PCa patients. Autophagy-related biomarkers have been implicated in the diagnosis and treatment of PCa [20].

LncRNAs are engaged in numerous biological processes, including epigenetic modulation, imprinting, and transcription [21]. Some lncRNAs can affect post-transcriptional regulation by interfering with microRNA. Besides, some lncRNAs can control gene expression at the epigenetic, transcriptional, and post-transcriptional levels [22–25]. Moreover, several lncRNAs have been shown to be effective biomarkers for PCa risk prediction with high sensitivity and specificity [26]. For example, a four-lncRNA signature was developed and used to predict biochemical recurrence-free survival in PCa patients, which will assist in the identification of high-risk patients who require more aggressive therapeutic interventions [27]. Importantly, lncRNAs can modulate autophagy in the malignant tumor by regulating targeted gene expression, and lncRNAs that modulate autophagy are vital in signal transduction and the underlying molecular mechanism in cancer [28]. Therefore, autophagy-related lncRNAs may be used as novel diagnostic and therapeutic markers for the PCa. In the current study, we focused on screening for lncRNAs that might play a pivotal role in the PCa by establishing a ceRNA regulatory network and constructing a signature of autophagy-related lncRNA for the diagnosis and prognosis of PCa patients.

The ceRNAs, acting as miRNA sponges, compete for miRNA binding sites and competitively regulate mRNAs using shared miRNA response elements in ceRNA networks. Moreover, the ceRNAs are all transcripts that may become miRNA targets, including non-coding long RNA (lncRNA), pseudogenic RNA, and circular RNA. The competing endogenous RNAs (ceRNA) hypothesis indicated that ceRNAs could indirectly modulate gene expression through competitive miRNA binding [29, 30]. However, studies on lncRNA-mediated ceRNA regulatory networks in PCa are insufficient. In this study, the analysis of DEmRNA, DEmiRNA, and DElncRNA between PCa and paracancerous tissues used the TCGA-PRAD data. Moreover, we have established a PCa-specific ceRNA regulatory network in PCa by integrating the interaction between autophagy-related DEmRNAs and DEmiRNAs or DEmiRNAs and DElncRNAs. Additionally, GEO data confirmed that the seven lncRNAs (ADAMTS9-AS1, ADAMTS9-AS2, MAGI2-AS3, PCA3, PCAT1, PVT1, and SNHG3) were expressed differentially in the ceRNA network, and these seven lncRNAs are related to autophagy in PCa. PCA3, as one of the selected autophagy-related DElncRNAs, expressed highly in PCa cells and tissues and has been identified and applied in clinical practice as a molecular marker of prostate cancer [31]. PCAT1 regulated the expression of CENPF, ID1, and ID3 in the cell cycle and proliferation, and promoted tumorigenesis in PCa by modulating FSCN1 via miR-145-5p [32, 33]. ADAMTS9-AS1 functioned as a ceRNA, a sponge for the hsa-mir-96, and a supportive regulator of PRDM16 expression in PCa [34]. MAGI2-AS3 was used in a nine-RNA signature for PCa prognosis through co-expression network analysis [35]. Then, the abnormally expressed PVT1 functioned as an oncogene in PCa, contributing to tumor growth [36]. Additionally, SNHG3 interactd with miR-577 to enhance SMURF1 expression in PCa [37]. Besides, ADAMTS9-AS2 is a new marker with no previous studies in PCa. Most of the lncRNAs examined in this study have only a small amount of research, and this study has laid a strong foundation for further exploring the function of lncRNA in autophagy PCa.

The majority of previous studies have concentrated on the function of genes involved in autophagy [38, 39]. There are currently no research that use autophagy-associated lncRNA signatures to predict PCa patient survival outcomes. Hence, it is necessary to establish autophagy-related lncRNA signatures to predict the prognosis of PCa patients. In this study, we obtained an autophagy-related lncRNA signature via Lasso regression and Cox regression, which is useful in the diagnosis and prognosis of PCa. Besides, the autophagy-associated lncRNA signature is an independent predictor of PCa, suggesting that the autophagy-associated lncRNA signature may be a prognostic biomarker for PCa patients. Additionally, there is currently a lack of research on the two autophagy-related lncRNAs (INE1 and MKNK1-AS1) in PCa. More research is therefore needed to explore how these lncRNAs affect the prognosis of PCa patients through autophagy.

This research contains certain drawbacks. Firstly, our data is primarily derived from publicly available databases, and the clinical implementation of our results necessitates further investigation. Secondly, we have not further experimentally verified the function of the screened lncRNAs. Finally, the signature requires external validation.

In conclusion, we developed a ceRNA regulatory network to investigate the lncRNAs that could play a key role in PCa autophagy, and we tested the differential expression of the selected lncRNAs using multiple GEO datasets. In addition, we successfully constructed an autophagy-related lncRNA signature for the diagnosis and prognosis of PCa patients, which could help with clinical decisions.

## MATERIALS AND METHODS

### Patient data extraction

Data on RNA-sequencing (RNA-seq) and corresponding clinical information were derived from The Cancer Genome Atlas Prostate Adenocarcinoma (TCGA-PRAD, https://portal.gdc.cancer.gov/) [40]. The criteria for excluding cases were developed as follows: (1) patient follow-up lasted less than 30 days; (2) patients received medication before resection; (3) the histological type of the patients did not belong to prostate adenocarcinoma acinar type; (4) Patient clinical information or RNA-seq data is not available.

### DEmRNAs, DEmiRNAs, and DElncRNAs in PCa

The differentially expressed mRNAs (DEmRNAs), differentially expressed miRNAs (DEmiRNAs), and DElncRNAs between PCa tissues and paracancerous tissues were analyzed using the limma package of R 4.0.2 software https://www.r-project.org/). |logFC|> 2, and the P -value< 0.05 were defined as thresholds. Furthermore, the gplots package in R 4.0.2 was applied to obtain volcano plots and heatmaps.

### Establishment of a ceRNA regulatory network

The interaction between autophagy-associated DEmRNAs and DEmiRNAs was predicted using the miRTarBase [41], miRDB [42], and the TargetScan database [43]. In addition, the interaction between DEmiRNAs and DElncRNAs was matched by the miRcode database [44]. A ceRNA regulatory network was constructed to visualize the interaction between the selected miRNAs and mRNAs or lncRNAs associated with autophagy by Cytoscape (version 3.7.2) [45].

### Verification of hub lncRNAs in GEO database

Gene Expression Omnibus (GEO, https://www.ncbi.nlm.nih.gov/geo/) database datasets GSE21034 and GSE94767 were selected to check the hub lncRNAs [46].

### Selection of autophagy-associated lncRNAs

Two hundred and thirty-two genes linked to autophagy were derived from HADb (Human Autophagy Database, http://www.autophagy.lu/clustering/) [47]. Autophagy-related lncRNAs were also determined based on Pearson correlation coefficients between the expression of lncRNAs and autophagy-related genes. The absolute value of the correlation coefficient > 0.5 and the P-value < 0.05 were applied to select the lncRNA related to autophagy.

### Identification of the autophagy-associated lncRNA signature

Univariate Cox regression and the Kaplan-Meier approach were also utilized to screen for autophagy-associated lncRNAs with P <0.05. Then, the obtained lncRNAs were included in the least absolute shrinkage and selection operator (LASSO) regression. Subsequently, a multivariate Cox regression was further used to construct an autophagy-related lncRNA signature to predict prognosis in PCs [48]. Finally, a survival analysis was performed to estimate the prognostic value of the screened lncRNAs using the survival package in R 4.0.2.

### Assessment of the autophagy-associated lncRNA signature

Based on the multivariate Cox regression's prognostic risk score, PCa patients were divided into high-risk and low-risk groups. The Kaplan–Meier survival curve and log-rank tests were applied to compare the differences in survival between high-risk and low-risk groups. The time-dependent receiver operating characteristic (ROC) curve and C-index calculation based on risk scores were conducted to assess the prognostic signature by the timeROC package and the survival package in R 4.0.2, respectively. Besides, the univariate and multivariate Cox regression analysis based on the prognostic signature and clinicopathological characteristics were further utilized to assess the independent prognostic factors for survival. Subsequently, the ROC curve was obtained by survivalROC package in R 4.0.2 to evaluate the relationship between clinicopathological features and the prognostic signature.

### Gene set enrichment analysis

The TCGA-PRAD samples were divided into high-risk and low-risk groups based on the risk scores of the lncRNA signature. Gene set enrichment analysis (GSEA, http://www.broadinstitute.org/gsea/index.jsp) was conducted to identify the signaling pathways and biological processes [49]. The nominal (NOM) p-value <0.05 and false discovery rate (FDR) <25% were deemed noteworthy.

### Statistical analysis

The data were processed and analyzed using the Perl data language (Version 5.30.2, http://www.perl.org) and R software (Version 4.0.2, https://www.r-project.org/). The survival curves were determined using the Kaplan-Meier method. The log-rank test was conducted to evaluate statistical differences between the high-risk and low-risk groups. A chi-squared test was used to investigate the relationship between risk scores and clinical features. The statistical tests were bilateral, and P <0.05 was considered statistically significant for all analyses.
